# Variation in clinical care associated with weekend admission and discharge in psychiatric in-patient units: retrospective case-note review

**DOI:** 10.1192/bjo.2020.88

**Published:** 2020-09-03

**Authors:** Ryan Williams, Lorna Farquharson, Ellen Rhodes, Mary Dang, Natasha Lindsay, Alan Quirk, David S. Baldwin, Mike J. Crawford

**Affiliations:** Department of Brain Sciences, Imperial College London; and College Centre for Quality Improvement, Royal College of Psychiatrists, London, UK; Department of Clinical Psychology, University of East London; and College Centre for Quality Improvement, Royal College of Psychiatrists, London, UK; College Centre for Quality Improvement, Royal College of Psychiatrists, London, UK; College Centre for Quality Improvement, Royal College of Psychiatrists, London, UK; College Centre for Quality Improvement, Royal College of Psychiatrists, London, UK; College Centre for Quality Improvement, Royal College of Psychiatrists, London, UK; Clinical and Experimental Sciences Academic Unit, University of Southampton; and College Centre for Quality Improvement, Royal College of Psychiatrists, London, UK; Department of Brain Sciences, Imperial College London; and College Centre for Quality Improvement, Royal College of Psychiatrists, London, UK

**Keywords:** Depressive disorders, in-patient treatment, anxiety disorders, quality of care, weekend

## Abstract

**Background:**

Questions have been raised regarding differences in the standards of care that patients receive when they are admitted to or discharged from in-patient units at weekends.

**Aims:**

To compare the quality of care received by patients with anxiety and depressive disorders who were admitted to or discharged from psychiatric hospital at weekends with those admitted or discharged during the ‘working week’.

**Method:**

Retrospective case-note review of 3795 admissions to in-patient psychiatric wards in England. Quality of care received by people with depressive or anxiety disorders was compared using multivariable regression analyses.

**Results:**

In total, 795 (20.9%) patients were admitted at weekends and 157 (4.8%) were discharged at weekends. There were minimal differences in quality of care between those admitted at weekends and those admitted during the week. Patients discharged at weekends were less likely to be given sufficient notification (48 h) in advance of being discharged (OR = 0.55, 95% CI 0.39–0.78), to have a crisis plan in place (OR = 0.65, 95% CI 0.46–0.92) or to be given medication to take home (OR = 0.45, 95% CI 0.30–0.66). They were also less likely to have been assessed using a validated outcome measure (OR = 0.70, 95% CI 0.50–0.97).

**Conclusions:**

There is no evidence of a ‘weekend effect’ for patients admitted to psychiatric hospital at weekends, but the quality of care offered to those who were discharged at weekends was relatively poor, highlighting the need for improvement in this area.

Concerns have been raised that the quality of in-patient care that people receive may vary according to the day of the week.^1^ Clinical outcomes may be worse among patients who are admitted to^2–7^ and discharged from^8^ acute hospitals at the weekend compared with those admitted and discharged during the ‘working week’.

Reasons for this ‘weekend effect’ are unclear. Although it has been suggested that increased mortality may be the result of lower staffing levels or poorer access to pathology, radiology and other services, others have noted that the ‘threshold’ for hospital admission may be higher at weekends and argued that poorer outcomes among those admitted over the weekend may be because their health problems are more severe.^9^ However, a recent meta-analysis found evidence of a weekend effect even after accounting for severity of disease.^10^

Studies to date have largely examined general hospitals providing acute medical, surgical and obstetric care: little research has been carried out in psychiatric hospitals, where there are over 100 000 in-patient admissions per year in England alone.^11^ The lack of research in this area is concerning, particularly as the periods immediately following admission and discharge have been identified as high-risk windows for adverse incidents in psychiatric in-patient units.^12,13^

One of the few studies in mental health services investigated mortality due to suicide, and found a ‘reverse’ weekend effect, whereby in-patients who died by suicide during an admission were less likely to have been admitted at the weekend.^14^ However, suicide during admission is a rare event and there are limitations to using this measure to evaluate quality of care.^15^ Another study reported shorter lengths of stay among those admitted at weekends,^16^ but this was conducted within a single organisation; the impact of weekend admission and discharge across a range of services has not been explored.

We therefore aimed to investigate whether weekend admission or discharge from psychiatric hospital was associated with worse clinical care for a specific patient cohort (those diagnosed with depressive illness, anxiety or stress-related disorders), using primary outcome measures based on National Institute for Health and Care Excellence (NICE) guidance for in-patient services^17–19^ and the Royal College of Psychiatrists’ *Standards for Inpatient Mental Health Services*.^20^

## Method

### Setting and participants

Data were obtained from the National Clinical Audit of Anxiety and Depression (NCAAD) carried out by the Royal College of Psychiatrists (RCPsych) in England in 2017–2018. The methodology for the audit has already been published and is available online.^21^ All in-patient mental health facilities in England that receive funding from the National Health Service (NHS) and provide services to adults diagnosed with anxiety and/or depressive disorders (54 trusts in total) were asked to take part. Restricting the sample to people with anxiety and depressive disorders reduced the impact of a potential confounder, that individuals admitted or discharged on weekends and on weekdays may have different clinical characteristics.

All services that took part in the audit were asked to supply an anonymous register of eligible patients who had been admitted to hospital during a sampling period from 1 April 2017 to 30 September 2017. If a patient had been admitted more than once during this sampling period, only the first admission was examined for the audit.

Patients were considered eligible for inclusion if they fulfilled the following criteria: age ≥16 years; and a recorded primary diagnosis of either an anxiety or a depressive disorder (as per ICD-10) at the point of discharge.

Patients were excluded if they had been given a primary diagnosis of bipolar affective disorder, cyclothymia, mania or any psychotic disorder during the admission. Those who were subsequently admitted to forensic or long-stay (e.g. rehabilitation) wards were also excluded.

If a service's register included >100 eligible cases, the RCPsych audit team selected 100 of these at random for inclusion in the audit.

### Data collection

All organisations that had been invited to participate (representing services provided by 54 NHS trusts) submitted data for the audit. Staff from the audit department of each organisation were asked to review the case notes for each of their eligible patients and complete an online data collection tool, using data from clinical records only. For each organisation, five of the sampled cases were selected at random for dual auditing (the tool was completed twice independently by separate auditors). For these cases, the two corresponding sets of results were then specifically examined by the RCPsych team to determine interrater reliability. Levels of interrater agreement were generally high, with 30% of items having complete agreement, 39% having substantial agreement and 31% having moderate to low agreement. In addition, three organisations were randomly selected for a quality assurance process that involved the RCPsych team visiting, auditing a random selection of cases directly and comparing these with the data that had been submitted – thereby ensuring that the results that had been submitted were accurate.

The audit tool was designed using guidance for in-patient services produced by NICE^17–19^ as well as *Standards for Inpatient Mental Health Services*, produced by the RCPsych's College Centre for Quality Improvement.^20^ It was formulated using input from psychiatric service providers, and public, patient and carer involvement groups. It included items examining patient demographics, characteristics of admission (time and date of admission and discharge), assessment (including physical health assessment), care planning, medication management, psychological therapies, crisis planning, discharge, follow-up and readmission. Before the main audit commenced, six volunteer trusts were enlisted in a pilot programme and completed an abbreviated version of the audit. The tool was refined further with their feedback, to guarantee that the audit process was easy to understand and practically achievable with the supporting information available.

The National Research Ethics Service and the Ethics and Confidentiality Committee of the National Information Governance Board were consulted, and they recommended that the project could be completed without formal ethical approval/written consent from participants because of the project's status as an audit (rather than a research project) and because patient-identifiable data were not being recorded. All procedures contributing to this work comply with the ethical standards of the relevant national and institutional committees on human experimentation and with the Helsinki Declaration of 1975, as revised in 2008.

### Exposure, outcome measures and covariates

‘Weekend admission’ was defined as being admitted to hospital between 00:00 h and 23:59 h on a Saturday, Sunday or UK public holiday. ‘Weekend discharge’ was defined as the end of a hospital admission taking place within that same time frame. The primary outcome measures were 23 items on quality of clinical care, based on NICE national guidelines^17–19,22^ and *Standards for Inpatient Mental Health Services* as defined by the RCPsych's College Centre for Quality Improvement.^20^

These were:

1. Did the (initial) assessment include details about the patient's past response to treatment?
2. Did the (initial) assessment consider whether the patient had a history of trauma?
3. Was there a documented current BMI?
4. Was there a documented current smoking status?
5. Was the identified family member, friend or carer provided with information about available support services and/or a support plan? (where an appropriate family member, friend or carer had been identified)
6. Was the identified family member, friend or carer offered a carer's assessment? (where an appropriate family member, friend or carer had been identified)
7. Did the patient have a care plan?
8. Is there evidence that the care plan was jointly developed between the patient and clinician?
9. Was the patient given a copy of their care plan?
10. Was the patient referred to psychological therapy?
11. Was the patient given at least 24 h notice of discharge?
12. Was the identified family member, friend or carer given at least 24 h notice of discharge? (where an appropriate family member, friend or carer had been identified)
13. Was the patient being prescribed psychotropic medication at the point of discharge?
14. Was the patient given verbal and/or written information about their medication prior to discharge?
15. Did a review of the patient's medication(s) take place prior to discharge?
16. At discharge, was the patient given ‘to take out/home’ (TTO) medication?
17. Did the patient have a crisis plan at the point of discharge?
18. Was a discharge letter sent to the patient's general practitioner within 24 h?
19. Was a care plan sent to a nominated person in an accepting service? (where an appropriate service had been identified)
20. Did the patient receive follow-up within 48 h of discharge?
21. Did a review of the patient's medication(s) take place between discharge and the end of the audit period?
22. Was an appropriately validated outcome measure completed?
23. Was the patient readmitted to hospital between discharge and the end of the audit period?

Covariates were also recorded – primary and secondary diagnoses, age, gender, ethnicity, employment status, accommodation status, wait time for bed, length of admission and detention status (whether admitted subject to restrictions imposed under the UK Mental Health Act 1983). The audit tool has been published online.^23^

### Statistical methods

We used SPSS for Windows^24^ to analyse study data. Initially, we calculated the proportion of patients who were admitted on each day of the week, and divided these into ‘weekend admissions’ and ‘weekday admissions’. This was repeated for discharges. Using univariate logistic regression, we examined the association of covariates (primary and secondary diagnosis, age, gender, ethnicity, employment and accommodation status, mode of admission) with weekend admission. We then used binomial logistic regression to examine the association of weekend admission with each primary outcome measure. This process was repeated for discharges, omitting two items that related only to initial assessment, as these were not judged to be relevant for weekend discharges.

As patients were clustered by service (i.e. quality of care for patients treated in the same service may be more similar compared with those treated in another service), all analyses were adjusted using multilevel logistic regression. Initially, the association between weekend admission/discharge and each quality of care variable was examined without accounting for any confounding variables. We then performed each analysis again, adjusting for the effects of service-level variation as well as any other variables that had been found to be significantly associated with the primary outcome measure (e.g. patient demographics such as age, gender, etc.).

## Results

**Fig. 1:**
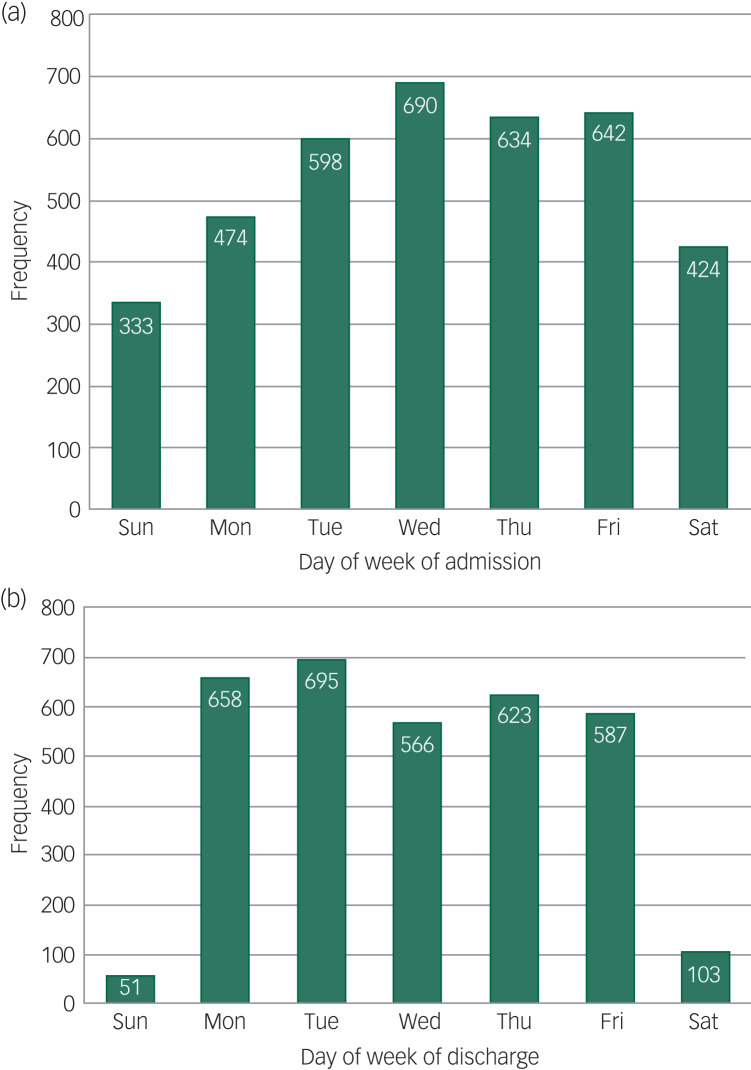
Psychiatric unit admissions (a) and discharges (b) by day of week.

In total, 54 NHS trusts participated in the audit, examining the case notes for 3795 patients. A total of 795 admissions (20.9%) took place at weekends (including public holidays) and 157 discharges (4.8%) were at weekends (Fig. 1).

Supplementary Tables 1 and 2, available at https://doi.org/10.1192/bjo.2020.88, compare the demographic and clinical characteristics of patients who were admitted and discharged at weekends with those of patients admitted and discharged during the working week. Patients below the age of 18 were less likely than all other age groups to be admitted at weekends; homeless patients were more likely to be admitted and discharged at weekends.

Neither weekend admission nor discharge was associated with particular primary or secondary diagnoses. Univariate analysis indicated an association between weekend admission and employment status (those admitted at weekends had shorter admissions than those admitted during the week) but there was no evidence for these effects after adjustment for other factors.

**Fig. 2:**
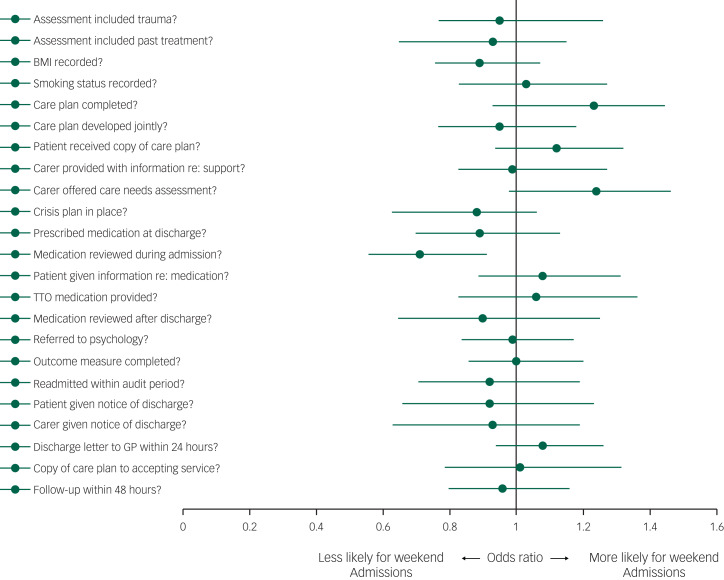
Forest plot of association between weekend psychiatric hospital admission and quality of care measures. TTO, to take out/home; GP, general practitioner.

Figure 2 and supplementary Table 3 show the results of multivariate regression analyses – displaying the effect of weekend admission on the primary outcome measures. There were few differences in these measures between patients admitted during weekends and those admitted during the week. Patients who were admitted at weekends were less likely to receive a medication review during their admission (OR = 0.71, 95% CI 0.56–0.91, *P* = 0.005), but there were no significant differences between groups in any of the other outcome measures. Univariate analysis suggested that patients admitted at weekends were less likely to be provided with medication to take home following discharge, but this association did not persist after adjustment for demographic and clinical factors.

**Fig. 3:**
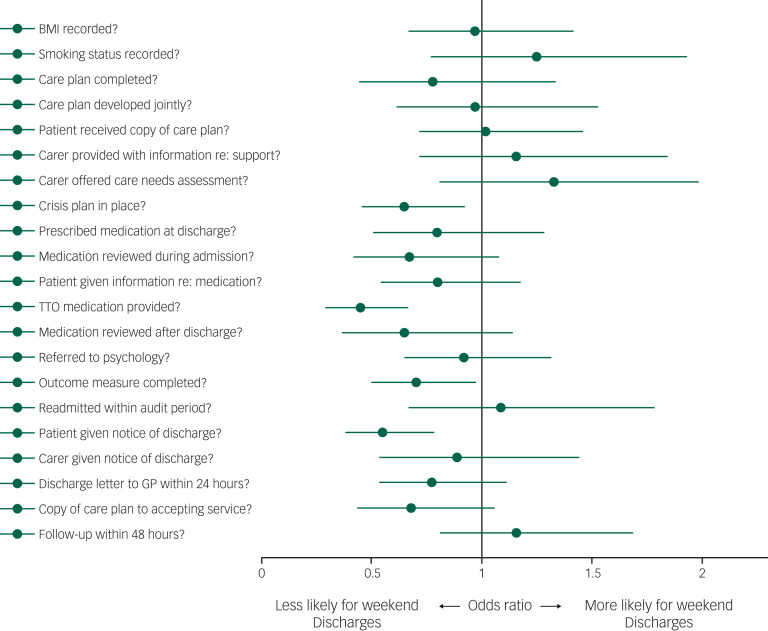
Forest plot of association between weekend psychiatric hospital discharge and quality of care measures. TTO, to take out/home; GP, general practitioner.

Figure 3 and supplementary Table 4 summarise the multivariate regression analyses investigating the association between weekend discharge and the primary outcome measures. Patients who were discharged at weekends were less likely to have received sufficient (at least 48 h) prior notification before being discharged (OR = 0.55, 95% CI 0.39–0.78, *P* = 0.001), less likely to have had a crisis plan in place at discharge (OR = 0.65, 95% CI 0.46–0.92, *P* = 0.014) and less likely to have been prescribed medication to take home with them (OR = 0.45, 95% CI 0.30–0.66, *P* < 0.0001). Patients discharged at weekends were also less likely to have been assessed using a validated outcome measure (OR = 0.70, 95% CI 0.50–0.97, *P* = 0.032).

## Discussion

Data from this study corroborate previous findings that patient turnover in psychiatric hospitals is reduced during weekends.^16^ Discharges are particularly affected, with only 4.7% taking place at weekends, while 19.9% of admissions take place at weekends. These findings demonstrate some variation in practice over the course of the week.

Young people aged 16–17 were considerably less likely to be admitted to hospital at weekends, which may reflect a deliberate strategy to avoid weekend admissions for this age group. Homeless individuals were more likely to be admitted and discharged from psychiatric hospital at weekends, suggesting that mechanisms designed to prevent patient turnover at weekends are less effective when patients lack stable accommodation or possibly a consistent point of contact with mental health services. After adjusting for demographic and clinical characteristics, we did not replicate a previous finding^16^ that females and individuals from ethnic minorities were more likely to be admitted at weekends, or that compulsory admission was less frequent at weekends. We also found no evidence that individuals with specific diagnoses were more likely to be admitted or discharged on a weekend.

We found minimal variation in quality of care between patients admitted at weekends and those admitted during the working week. By contrast, there was clear evidence for diminished quality of care among patients who were discharged at weekends. Aspects that differed related largely to the discharge planning process: patients discharged at weekends were less likely to have received sufficient prior notification before being discharged, to have had a crisis plan in place at the point of discharge and to have been prescribed medication to take away at the point of discharge.

### Strengths and limitations

A large sample was obtained, encompassing data from services providing acute psychiatric in-patient care in every NHS trust across England. We anticipate, therefore, that the sample is representative of the target population, and that the findings are generalisable to wider clinical practice relating to patients with anxiety and depressive disorders.

Prior to this investigation, the most comprehensive consideration of the weekend effect in psychiatric services^16^ was restricted to a single NHS trust, and used relatively rare serious events (in-patient mortality and violent incidents) to examine potential differences in care. By contrast, the primary outcome measures we used were based on NICE guidance^17–19^ and RCPsych standards,^20^ developed with input from an advisory group of providers and patient and carer representatives.

There are significant limitations. A retrospective case-note audit depends on accurate clinical records, which may fail to fully capture patient/carer experiences. By restricting the study to patients with diagnoses of anxiety and depressive disorders, we addressed a potential confounding effect identified in previous studies^14,16^ but the findings may not be generalisable to all people admitted to mental health units, in which the majority of psychiatric admissions relate to people with psychoses.

We were unable to gather information from individual services regarding staffing levels and operating procedures at weekends, as well as availability of community services (e.g. crisis/home treatment teams) and housing/Social Services. Finally, the cross-sectional design means that we are unable to examine and characterise temporal associations between weekend admission/discharge and standards of care: for example, although difficulties with the process of undertaking a discharge at the weekend might lead to poor care standards, equally poor care standards might lead to unplanned discharge during the weekend (e.g. by the patient discharging themselves).

### Implications

Strategies employed by the NHS in general hospitals to reduce or eliminate the weekend effect have focused on improving staffing levels, as reduced weekend staffing has been repeatedly highlighted as a major contributory factor, despite limited evidence.^25^ Mental health services also tend to operate with reduced scheduled activity and lower staffing at weekends, including limited input from senior clinicians.

Our findings provide little evidence for variation in quality of care for patients admitted to psychiatric hospitals at weekends. The absence of weekend variation relating to admission in this study may reflect the fact that some of the other possible causes of a weekend effect in general hospitals – such as lack of access to specialist equipment or investigations^26^ – are less applicable to mental health settings.

We found some evidence that quality of care was worse for patients who were discharged from psychiatric hospital at weekends. A small minority of discharges took place at weekends, and it is hard to know whether quality of care was affected because of inherent difficulties with the discharge process at weekends (such as lack of access to pharmacy services, absence of input from senior clinicians, and reduced availability of community mental health and Social Services).

These findings merit further research to identify the exact reasons for the relatively poor quality of care for weekend discharges. Discharge planning is a crucial stage of any hospital admission, and a poorly planned or executed discharge may undermine the efficacy of a period of in-patient treatment. Research with patients has found that discharge is often experienced as chaotic and distressing,^27^ and rates of adverse incidents such as self-harm and suicide are particularly high in the period immediately following discharge from psychiatric hospital.^28,29^ Unplanned discharge has been associated with greater risk of suicide,^30^ and discharge planning interventions have been shown to be effective in improving outcomes, including reducing readmission and improving adherence to aftercare.^31^

Although we did not find differences in the quality of care of patients admitted to mental health units during the working week and at weekends, it is important to note that the quality of care that patients received (regardless of when they were admitted) fell short of national standards. As an example, <60% of patients had been followed up within 48 h of discharge, or had a discharge letter sent to their general practitioner within 24 h of discharge, and <30% of carers were offered an assessment of their needs. These findings will be addressed in the forthcoming NCAAD core audit report, suggested as potential targets for quality improvement activities by the RCPsych and re-examined in future cycles of the national audit.

## Data Availability

The data-set is held by the NCAAD team at the Royal College of Psychiatrists’ College Centre for Quality Improvement and could be made available on request. All authors had access to the full study data-set.

## Supplementary material

For supplementary material accompanying this paper visit http://dx.doi.org/10.1192/bjo.2020.88. click here to view supplementary material
