# Supplementary material for: Variation in clinical care associated with weekend admission and discharge in psychiatric in-patient units: retrospective case-note review
Source: BJPsych Open. 2020 Sep 3;6(5):e103. doi: 10.1192/bjo.2020.88 (PMC7488328; doi:10.1192/bjo.2020.88)
Supplement: Supplementary file 1 [file bjosup.zip › S2056472420000885sup002.docx]

## Table 1

| Table 1 - Analysis of demographic / clinical factors associated with weekend psychiatric hospital admission | | | | | | |
| --- | --- | --- | --- | --- | --- | --- |
|  | WeekDAY admission  N  (% of row) | WeekEND admission  N  (% of row) | Unadjusted OR  (95% CI) | P | Adjusted^a^ OR  (95% CI) | P |
| Age  n=3795 |  |  |  |  |  |  |
| <18 | 85  (89.5) | 10  (10.5) | 0.40  0.20 to 0.79 | 0.008 | 0.32  0.11 to 0.94 | 0.038 |
| 18 to 24 | 316  (77.6) | 91  (22.4) | 0.98  0.74 to 1.32 | 0.912 | 1.19  0.85 to 1.69 | 0.299 |
| 25 to 34 | 539  (78.5) | 148  (21.5) | 0.94  0.73 to 1.21 | 0.620 | 1.03  0.78 to 1.37 | 0.834 |
| 35 to 44 | 465  (76.0) | 147  (24.0) | 1.08  0.84 to 1.39 | 0.555 | 1.07  0.80 to 1.44 | 0.635 |
| 45 to 54 | 550  (77.4) | 161  (22.6) | Ref |  | Ref |  |
| 55 to 64 | 393  (78.0) | 111  (22.0) | 0.97  0.73 to 1.27 | 0.798 | 0.89  0.64 to 1.24 | 0.479 |
| 65 to 74 | 351  (83.6) | 69  (16.4) | 0.67  0.49 to 0.92 | 0.012 | 0.71  0.41 to 1.21 | 0.206 |
| 75+ | 301  (83.8) | 58  (16.2) | 0.66  0.47 to 0.92 | 0.013 | 0.69  0.39 to 1.23 | 0.211 |
| Gender  n=3789 |  |  |  |  |  |  |
| Male | 1531  (78.8) | 413  (21.2) | Ref |  | Ref |  |
| Female | 1463  (79.3) | 382  (20.7) | 0.97  0.83 to 1.13 | 0.683 | 1.09  0.91 to 1.30 | 0.366 |
| Ethnicity  n=3565 |  |  |  |  |  |  |
| White | 2531  (79.2) | 663  (20.8) | Ref |  | Ref |  |
| Black | 63  (77.8) | 18  (22.2) | 1.09  0.64 to 1.85 | 0.748 | 1.13  0.65 to 1.96 | 0.676 |
| Asian | 120  (78.9) | 32  (21.2) | 1.02  0.68 to 1.52 | 0.930 | 0.81  0.52 to 1.27 | 0.355 |
| Mixed | 52  (75.4) | 17  (24.6) | 1.25  0.72 to 2.17 | 0.433 | 1.05  0.57 to 1.94 | 0.875 |
| Other | 55  (79.7) | 14  (20.3) | 0.97  0.54 to 1.76 | 0.924 | 0.88  0.45 to 1.73 | 0.713 |
| Employment  n=3305 | |  |  |  |  |  |
| Unemployed | 639  (75.1) | 212  (24.9) | Ref |  | Ref |  |
| Employed | 677  (78.7) | 183  (21.3) | 0.82  0.65 to 1.02 | 0.075 | 0.84  0.66 to 1.07 | 0.167 |
| Long-term sick | 527  (79.2) | 138  (20.8) | 0.79  0.62 to 1.01 | 0.057 | 0.78  0.60 to 1.01 | 0.063 |
| Retired | 641  (83.0) | 131  (17.0) | 0.62  0.48 to 0.79 | <0.001 | 0.86  0.53 to 1.40 | 0.533 |
| Student | 136  (86.6) | 21  (13.4) | 0.47  0.29 to 0.76 | 0.002 | 0.60  0.32 to 1.12 | 0.106 |
| Accommodation  n=3508 | |  |  |  |  |  |
| Mainstream | 2332  (79.4) | 604  (20.6) | Ref |  | Ref |  |
| Supported | 139  (74.7) | 47  (25.3) | 1.31  0.93 to 1.84 | 0.127 | 1.211  0.85 to 1.74 | 0.297 |
| Homeless | 160  (71.7) | 63  (28.3) | 1.52  1.12 to 2.06 | 0.007 | 1.51  1.04 to 2.18 | 0.031 |
| Other | 135  (82.8) | 28  (17.2) | 0.80  0.53 to 1.22 | 0.296 | 0.81  0.51 to 1.29 | 0.374 |
| Detention Status  n = 3795 | |  |  |  |  |  |
| Informal | 2499  (79.0) | 666  (21.0) | Ref |  | Ref |  |
| Formal | 501  (79.5) | 129  (20.5) | 0.65  0.40-1.08 | 0.094 | 0.70  0.40 to 1.25 | 0.231 |
|  | WeekDAY admission  Mean  Median  STD | WeekEND admission  Mean  Median  STD | Unadjusted  B coefficient  95% CI | P | Adjusted^a^  B coefficient  95% CI | P |
| Wait time for bed  n=2207 | 33.01 hours  5.72 hours  58.13 hours | 23.04 hours  4.52 hours  68.54 hours | - 9.96 hours  - 25.58 to 5.65 hours | 0.211 | - 7.86 hours  - 20.61 to 4.89 hours | 0.227 |
| Length of admission n=3795 | 27.06 days  14.00 days  39.57 days | 23.54 days  11.00 days  40.04 days | - 3.51 days  - 6.86 to - 0.17 days | 0.039 | - 2.64 days  - 6.31 to 1.02 days | 0.158 |

1. Adjusted for NHS trust, age, employment/ accommodation status, primary/ secondary diagnosis, length of admission and detention status.

## Table 2

| Table 2 - Analysis of demographic / clinical factors associated with weekend psychiatric hospital discharge | | | | | | |
| --- | --- | --- | --- | --- | --- | --- |
|  | WeekDAY discharge  N  (% of row) | WeekEND discharge  N  (% of row) | Unadjusted OR  (95% CI) | P | Adjusted^b^ OR  (95% CI) | P |
| Age  n=3283 |  |  |  |  |  |  |
| <18 | 73  (94.8) | 4  (5.2) | 1.19  0.40 to 3.48 | 0.756 | 1.56  0.29 to 8.33 | 0.604 |
| 18 to 24 | 353  (94.6) | 20  (5.4) | 1.15  0.64 to 2.10 | 0.639 | 1.24  0.61 to 2.51 | 0.549 |
| 25 to 34 | 579  (94.0) | 37  (6.0) | 1.24  0.74 to 2.07 | 0.411 | 1.08  0.60 to 1.95 | 0.798 |
| 35 to 44 | 533  (96.4) | 20  (3.6) | 0.78  0.44 to 1.41 | 0.412 | 0.62  0.31 to 1.23 | 0.172 |
| 45 to 54 | 587  (95.1) | 30  (4.9) | Ref |  | Ref |  |
| 55 to 64 | 403  (94.8) | 22  (5.2) | 1.08  0.60 to 1.92 | 0.802 | 0.94  0.47 to 1.87 | 0.852 |
| 65 to 74 | 341  (96.9) | 11  (3.1) | 0.66  0.33 to 1.35 | 0.259 | 0.58  0.17 to 1.95 | 0.379 |
| 75+ | 257  (95.2) | 13  (4.8) | 0.97  0.49 to 1.94 | 0.933 | 0.86  0.25 to 2.98 | 0.810 |
| Gender  n=3277 |  |  |  |  |  |  |
| Male | 1626  (94.8) | 89  (5.2) | Ref |  | Ref |  |
| Female | 1495  (95.7) | 67  (4.3) | 0.80  0.57 to 1.12 | 0.193 | 1.02  0.69 to 1.50 | 0.939 |
| Ethnicity  n=3084 |  |  |  |  |  |  |
| White | 2620  (95.4) | 126  (4.6) | Ref |  | Ref |  |
| Black | 74  (97.4) | 2  (2.6) | 0.59  0.14 to 2.43 | 0.466 | 0.29  0.04 to 2.16 | 0.228 |
| Asian | 130  (96.3) | 5  (3.7) | 0.88  0.35 to 2.18 | 0.775 | 1.02  0.40 to 2.62 | 0.961 |
| Mixed | 59  (96.7) | 2  (3.3) | 0.38  0.05 to 2.75 | 0.337 | 0.39  0.05 to 2.88 | 0.357 |
| Other | 65  (98.5) | 1  (1.5) | 0.34  0.05 to 2.49 | 0.289 | 0.28  0.01 to 3.48 | 0.997 |
| Employment n=2867 |  |  |  |  |  |  |
| Unemployed | 702  (94.7) | 39  (5.3) | Ref |  | Ref |  |
| Employed | 752  (95.3) | 37  (4.7) | 0.89  0.56 to 1.43 | 0.640 | 0.77  0.45 to 1.33 | 0.344 |
| Long-term sick | 540  (94.2) | 33  (5.8) | 1.08  0.66 to 1.76 | 0.753 | 1.07  0.62 to 1.84 | 0.818 |
| Retired | 601  (96.2) | 24  (3.8) | 0.70  0.42 to 1.20 | 0.194 | 1.03  0.36 to 2.96 | 0.956 |
| Student | 134  (96.4) | 5  (3.6) | 0.71  0.27 to 1.83 | 0.473 | 0.63  0.17 to 2.31 | 0.485 |
| Accommodation n=3052 |  |  |  |  |  |  |
| Mainstream | 2427  (95.2) | 122  (4.8) | Ref |  | Ref |  |
| Supported | 155  (95.7) | 7  (4.3) | 0.81  0.35 to 1.87 | 0.618 | 0.69  0.25 to 1.93 | 0.690 |
| Homeless | 182  (90.5) | 19  (9.5) | 2.11  1.25 to 3.54 | 0.005 | 1.84  1.05 to 3.52 | 0.047 |
| Other | 137  (97.9) | 3  (2.1) | 0.47  0.15 to 1.48 | 0.196 | 0.42  0.10 to 1.75 | 0.235 |
| Detention Status  n = 3283 |  |  |  |  |  |  |
| Informal | 2608  (94.9) | 139  (5.1) | Ref |  | Ref |  |
| Formal | 518  (96.6) | 18  (3.4) | 0.69  0.42 to 1.15 | 0.153 | 0.73  0.41 to 1.30 | 0.283 |
|  | WeekDAY discharge  Mean  Median  STD | WeekEND discharge  Mean  Median  STD | Unadjusted  B coefficient  95% CI | P | Adjusted^b^  B coefficient  95% CI | P |
| Wait time  for bed  n=1981 | 31.52 hours  5.42 hours  49.09 hours | 18.94 hours  5.47 hours  40.05 hours | - 12.58 hours  - 42.80 to 17.64 hours | 0.415 | - 8.73 hours  - 33.45 to 15.98 hours | 0.488 |
| Length of admission n=3283 | 26.46 days  14.00 days  39.65 days | 22.91 days  8.00 days  40.53 days | - 3.55 days  - 10.08 to 2.98 days | 0.286 | - 1.35 days  - 8.66 to 5.97 days | 0.718 |

1. Adjusted for NHS trust, accommodation status, and primary/ secondary diagnosis.

## Table 3

| Table 3 – association of weekend psychiatric hospital admission with quality of care measures. | | | | | | |
| --- | --- | --- | --- | --- | --- | --- |
| Primary outcome | WeekDAY  admission  N / total  (%) | WeekEND  admission  N / total  (%) | OR  95% CI | P | Adjusted^a^ OR  95% CI | P |
| Assessment included trauma? | 2058/2618  (78.6) | 557/712  (78.2) | 0.98  0.80 to 1.20 | 0.827 | 0.95  0.77 to 1.16 | 0.609 |
| Assessment included past treatment? | 2132/2602  (81.9) | 544/685  (79.4) | 0.85  0.69 to 1.05 | 0.132 | 0.93  0.65 to 1.15 | 0.479 |
| BMI recorded | 2177/3000  (72.6) | 549/795  (69.1) | 0.84  0.71 to 1.00 | 0.051 | 0.89  0.76 to 1.07 | 0.222 |
| Smoking status recorded | 2493/3000  (83.1) | 662/795  (83.3) | 1.01  0.82 to 1.25 | 0.909 | 1.03  0.83 to 1.27 | 0.793 |
| Care plan completed | 2717/2999  (90.6) | 728/795  (91.6) | 1.13  0.85 to 1.49 | 0.398 | 1.23  0.93 to 1.44 | 0.144 |
| Care plan developed jointly | 2238/2717  (82.4) | 588/728  (80.8) | 0.90  0.73 to 1.11 | 0.318 | 0.95  0.77 to 1.18 | 0.660 |
| Patient received copy of care plan | 1513/2717  (55.7) | 420/728  (57.7) | 1.09  0.92 to 1.28 | 0.333 | 1.12  0.94 to 1.32 | 0.202 |
| Carer provided info re: support services available | 1162/1859  (62.5) | 296/480  (61.7) | 0.97  0.79 to 1.17 | 0.735 | 0.99  0.83 to 1.27 | 0.820 |
| Carer offered care needs assessment | 448/1859  (24.1) | 131/480  (27.3) | 1.18  0.94 to 1.48 | 0.149 | 1.24  0.98 to 1.46 | 0.076 |
| Crisis plan in place at discharge | 1940/2592  (74.8) | 508/708  (71.8) | 0.85  0.71 to 1.03 | 0.096 | 0.88  0.63 to 1.06 | 0.178 |
| Prescribed medication at discharge? | 2639/3000  (88.0) | 678/795  (85.3) | 0.79  0.63 to 0.99* | 0.043 | 0.89  0.70 to 1.13 | 0.338 |
| Medication reviewed during admission? | 2300/2612  (88.1) | 561/673  (83.4) | 0.68  0.54 to 0.86* | 0.001 | 0.71  0.56 to 0.91* | 0.005 |
| Patient given info re: medication? | 1917/2639  (72.6) | 499/678  (73.6) | 1.05  0.87 to 1.27 | 0.617 | 1.08  0.89 to 1.31 | 0.416 |
| TTA medication provided at discharge? | 1999/2360  (84.7) | 548/651  (84.2) | 0.96  0.76 to 1.22 | 0.742 | 1.06  0.83 to 1.36 | 0.629 |
| Medication reviewed after discharge? | 1349/1555  (86.8) | 339/396  (85.6) | 0.91  0.66 to 1.25 | 0.551 | 0.90  0.65 to 1.24 | 0.511 |
| Referred to psychology? | 1092/2999  (36.4) | 281/795  (35.3) | 0.96  0.81 to 1.12 | 0.578 | 0.99  0.84 to 1.17 | 0.932 |
| Outcome measure completed | 1832/3000  (61.1) | 482/795  (60.6) | 0.98  0.84 to 1.15 | 0.822 | 1.00  0.86 to 1.20 | 0.840 |
| Readmission within audit period | 328/2593  (12.5) | 84/708  (11.9) | 0.93  0.72 to 1.20 | 0.575 | 0.92  0.71 to 1.19 | 0.508 |
| Patient given notice of discharge | 2017/2593  (77.1) | 529/708  (74.7) | 0.84  0.70 to 1.02 | 0.085 | 0.92  0.66 to 1.23 | 0.429 |
| Carer given notice of discharge | 1119/1589  (70.4) | 282/422  (66.8) | 0.85  0.67 to 1.06 | 0.153 | 0.93  0.63 to 1.19 | 0.574 |
| Discharge letter sent to GP within 48 hours | 1164/2235  (52.1) | 319/598  (53.3) | 1.05  0.88 to 1.29 | 0.583 | 1.08  0.94 to 1.26 | 0.429 |
| Copy of care plan sent to accepting service | 1212/1608  (75.4) | 314/422  (74.4) | 0.95  0.74 to 1.22 | 0.683 | 1.01  0.79 to 1.31 | 0.895 |
| Follow-up within 48 hours of discharge | 1230/2231  (55.1) | 322/595  (54.1) | 0.96  0.80 to 1.15 | 0.659 | 0.96  0.80 to 1.16 | 0.680 |

1. Adjusted for NHS trust, age, employment/ accommodation status, primary/ secondary diagnosis, length of admission and detention status.

## Table 4

| Table 4 – association of weekend psychiatric hospital discharge with quality of care measures. | | | | | | | |
| --- | --- | --- | --- | --- | --- | --- | --- |
| Primary outcome | WeekDAY  discharge  N / total  (%) | WeekEND  discharge  N / total  (%) | OR  95% CI | P | | Adjusted^b^ OR  95% CI | P |
| BMI recorded? | 2248/3126 (71.9) | 113/157 (72.0) | 1.00  0.70 to 1.43 | 0.987 | | 0.97  0.67 to 1.41 | 0.886 |
| Smoking status recorded? | 2600/3126 (83.2) | 136/157 (86.6) | 1.31  0.82 to 2.09 | 0.259 | | 1.25  0.77 to 1.93 | 0.361 |
| Care plan completed? | 2844/3125 (91.0) | 138/157 (87.9) | 0.72  0.44 to 1.18 | 0.189 | | 0.78  0.45 to 1.33 | 0.358 |
| Care plan developed jointly? | 2322/2844 (81.6) | 113/138 (81.9) | 1.02  0.65 to 1.58 | 0.944 | | 0.97  0.62 to 1.52 | 0.903 |
| Patient received copy of care plan? | 1595/2844 (56.1) | 80/138 (58.0) | 1.08  0.76 to 1.53 | 0.663 | | 1.02  0.72 to 1.45 | 0.917 |
| Carer provided info re: support services available | 1194/1921 (62.2) | 53/81 (65.4) | 1.15  0.72 to 1.87 | 0.551 | | 1.16  0.72 to 1.84 | 0.549 |
| Carer offered care needs assessment | 469/1921 (24.4) | 25/81 (30.9) | 1.38  0.85 to 2.24 | 0.189 | | 1.33  0.81 to 1.98 | 0.256 |
| Crisis plan in place at discharge | 2330/3125 (74.6) | 102/157 (65.0) | 0.63  0.45 to 0.89** | 0.008 | | 0.65  0.46 to 0.92* | 0.014 |
| Prescribed medication at discharge? | 2739/3126 (87.6) | 132/157 (84.1) | 0.075  0.48 to 1.16 | 0.192 | | 0.80  0.51 to 1.28 | 0.355 |
| Medication reviewed during admission? | 2375/2734 (86.9) | 107/131 (81.7) | 0.67  0.43 to 1.06 | 0.090 | | 0.67  0.42 to 1.07 | 0.095 |
| Patient given info re: medication? | 2005/2739 (73.2) | 92/132 (69.7) | 0.84  0.58 to 1.23 | 0.376 | | 0.80  0.54 to 1.17 | 0.243 |
| TTA medication provided at discharge? | 2432/2854 (85.2) | 101/141 (71.6) | 0.44  0.30 to 0.64** | | <0.0001 | 0.45  0.30 to 0.66** | <0.0001 |
| Medication reviewed after discharge? | 1602/1847 (86.7) | 76/93 (81.7) | 0.68  0.40 to 1.18 | 0.170 | | 0.65  0.37 to 1.14 | 0.136 |
| Referred to psychology? | 1145/3125 (36.6) | 52/157 (33.1) | 0.86  0.061 to 1.20 | 0.372 | | 0.92  0.65 to 1.31 | 0.654 |
| Outcome measure completed | 1930/3126 (61.7) | 83/157 (52.9) | 0.69  0.50 to 0.95* | 0.027 | | 0.70  0.50 to 0.97* | 0.032 |
| Readmission within audit period | 391/3126 (12.5) | 21/157 (13.4) | 1.08  0.67 to 1.73 | 0.749 | | 1.09  0.67 to 1.78 | 0.719 |
| Patient given notice of discharge | 2431/3126 (77.8) | 101/157 (64.3) | 0.052  0.37 to 0.72** | <0.0001 | | 0.55  0.39 to 0.78** | 0.001 |
| Carer given notice of discharge | 1340/1921 (69.8) | 54/81 (66.7) | 0.87  0.054 to 1.39 | 0.554 | | 0.89  0.54 to 1.44 | 0.622 |
| Discharge letter sent to GP within 48 hours | 1411/2694 (52.4) | 57/124 (46.0) | 0.67  0.45 to 0.98* | 0.047 | | 0.77  0.54 to 1.11 | 0.064 |
| Copy of care plan sent to accepting service | 1447/1914 (75.6) | 70/103 (68.0) | 0.69  0.045 to 1.05 | 0.082 | | 0.68  0.44 to 1.05 | 0.083 |
| Follow-up within 48 hours of discharge | 1471/2685 (54.8) | 74/127 (58.3) | 1.15  0.80 to 1.65 | 0.441 | | 1.16  0.81 to 1.68 | 0.419 |

1. Adjusted for NHS trust, accommodation status, and primary/ secondary diagnosis.
